# Gearbox Tooth Cut Fault Diagnostics Using Acoustic Emission and Vibration Sensors — A Comparative Study

**DOI:** 10.3390/s140101372

**Published:** 2014-01-14

**Authors:** Yongzhi Qu, David He, Jae Yoon, Brandon Van Hecke, Eric Bechhoefer, Junda Zhu

**Affiliations:** 1 Department of Mechanical and Industrial Engineering, University of Illinois at Chicago, Chicago, IL 60607, USA; E-Mails: yqu5@uic.edu (Y.Q.); jyoon52@uic.edu (J.Y.); bvanhe2@uic.edu (B.V.H.); 2 Green Power Monitoring Systems, LLC, Essex Junction, VT 05452, USA; E-Mail: eric@gpms-vt.com; 3 Renewable NRG Systems, Hinesburg, VT 05461, USA; E-Mail: jz@renewablenrgsystems.com

**Keywords:** gearbox faults, diagnostics, acoustic emission sensor, vibration sensor

## Abstract

In recent years, acoustic emission (AE) sensors and AE-based techniques have been developed and tested for gearbox fault diagnosis. In general, AE-based techniques require much higher sampling rates than vibration analysis-based techniques for gearbox fault diagnosis. Therefore, it is questionable whether an AE-based technique would give a better or at least the same performance as the vibration analysis-based techniques using the same sampling rate. To answer the question, this paper presents a comparative study for gearbox tooth damage level diagnostics using AE and vibration measurements, the first known attempt to compare the gearbox fault diagnostic performance of AE- and vibration analysis-based approaches using the same sampling rate. Partial tooth cut faults are seeded in a gearbox test rig and experimentally tested in a laboratory. Results have shown that the AE-based approach has the potential to differentiate gear tooth damage levels in comparison with the vibration-based approach. While vibration signals are easily affected by mechanical resonance, the AE signals show more stable performance.

## 1. Introduction

Gearboxes are used in almost all transmission systems and power systems. Wind turbine systems and helicopter impetus systems are two typical systems that rely heavily on gearboxes. According to the existing literature, gear failure plays a critical role in the overall failure modes of a gearbox and transmission system. As reported in [1], approximately 59% of the failure modes in wind turbines involved gear failures. In another report [2], it was shown that among all the helicopter transmission system failures, 19.1% of them included gear failures. The use of a health and usage monitoring system (HUMS) is mandatory for helicopter operating service. HUMS allows better and more cost effective maintenance practices because it can provide indications and warnings prior to any collateral damages. Various sensing techniques have been applied to gearbox fault diagnostics, such as vibration, acoustic emission (AE), oil debris, and so on. Currently, vibration analysis is still the most widely used technique in industry. Acoustic emission has been investigated as a potential alternative for machinery fault detection and diagnosis.

Vibration analysis is a well-developed technique for machinery fault diagnosis. A large number of research papers on vibration-based fault detection and diagnosis have been published. When there is a force variation in a gearbox, the component will generate a vibration. This vibration is then transmitted to the surrounding structure, and therefore noise and vibration will be generated in the gearbox [3]. Transmission error (TE) is generally considered to be the primary excitation mechanism for gear noise and vibration. According to [4], a transmission error is defined as “the difference between the actual position of the output gear and the position it would occupy if the gear drive were perfectly conjugate”. Vibration signal analysis is an important tool to experimentally investigate gear vibration because gears generate vibrations at specific frequencies, which are related to the number of gear teeth and the rotational speed of the gear shaft.

AE is defined as transient elastic waves within a material caused by deformation and the release of localized stress energy [5]. Even though AE has been studied as a potential tool for machine fault diagnosis for a long time, the source and characteristics of AE signals, especially in machine fault detection, are still not fully understood. Initially, burst type AE signals were used for fault detection in structural health monitoring. The AE bursts are believed to be fault related. While this might hold a ground truth for static structural fault detection, it has never been proved for rotating machines. For bearings, it has been proposed that asperity contact was the primary sources of AE signals [6]. For gears, similar studies have not been performed systematically yet. The relationship between AE signals and asperity contact under an elastohydrodynamic lubrication regime has been studied, which is synonymous with gears [7,8]. The authors in [7] and [8] identified asperity contact as a significant source of AE signals, but did not investigate other sources such as the gear dynamics, backlash and so on in detail. It is generally accepted that an increase in meshing stress would generate AE responses with larger amplitude [9]. In this paper, AE signals are postulated to be mostly related to the interaction and impact of teeth during tooth meshing. The impact on the surface of the tooth causes material deformation and this is followed by the stress energy release, which will then cause transient elastic waves.

In gear and bearing fault diagnosis, research has reported that AE sensors are more sensitive to early faults than vibration sensors. For gears, Tandon and Mata [10] applied AE to a spur gears test rig with a jet oil lubrication system to investigate the detectability of gear pitting damages. Simulated pitting has constant depth (500 μm) but variable diameter (250/350/450/550/1,100 and 2,200 μm). Their investigation has shown the advantage of AE over vibration for early detection of defects in gears by observing that the AE data displayed a sharp increase in the parameters when the defect size was around 500 μm, while vibration data did not display a comparable increase until the defect size was more than 1,000 μm. Scheer *et al*. [11] have shown that AE is effective to capture early stages of gear faults (e.g., tooth edge fracture and pitting) before they grow and change their vibration behavior. For bearings, Yoshioka and Fujiwara [12,13] have shown that AE parameters were able to identify bearing defects before their appearance in the vibration range. This led to an investigation that used the AE technique for the detection of subsurface cracks resulting from rolling contact fatigue [14]. The method provided the ability to determine the position of sub-surface fatigue cracks by relating the crack positions to the location of the AE signal source. The conclusions in [12,13] were later validated by Hawman and Galinaitisin [15] in a study that also made the observation that AE techniques are able to detect bearing faults earlier than vibration analysis methods. In a study by Eftekharnejad *et al*. [16] comparing the applicability of AE and vibration technologies for the monitoring of rolling bearing degradation, it was shown that AE was more sensitive for incipient fault detection when compared to vibration.

AE signals are relatively unaffected by structural resonance and could be more sensitive to early fault activities [17]. When an unknown fault starts to form in the machinery, energy loss actions such as impacts, friction, and crushing generate sound wave activity that spans a broad range of frequencies [18]. AE sensors could capture frequencies that are much higher than those in vibration signals and therefore their use enables the technicians to detect inchoate faults before any damage occurs. Also, by quantitative methods, one could monitor the fault evolution process from the very beginning. Compared with vibration analysis, AE signals have the potential to detect small abnormal friction, initial cracking and so on. There are some possible explanations for this. The first one, as discussed above, is that AE emitted by very small defects occurs in frequency ranges that are higher than the operational ranges of vibration sensors and therefore might not be caught by vibration sensors. The second explanation is that when there is only a small crack or surface wear in the machinery, it is not severe enough to change the structural vibration. Vibration signals collected by accelerometers, which measures the second derivative of the displacement, may still remain the same, and thus be unable to detect the incipient fault. In this paper, vibration sensors are explicitly assumed as accelerometers.

Many studies on AE- and vibration-based gear fault detection have been reported. Ogbonnah [19] applied a wavelet analysis method to gear fault diagnosis and prognosis using AE sensors. A linear relationship between AE amplitude, gearbox running time, and pit progression was shown in that study. It has been shown that the wavelet analysis method offers good prognosis for the pitting progression as well as the pitting rate. In an early study which applied the AE technique to the analysis of fatigue crack growth in a carburized gear tooth [20], AE energy rate was found to be proportional to the stress intensity factor range and crack growth rate. Another comparative study using AE, vibration and spectrometric oil samples for spur gear pitting fault detection was reported in [7]. As an experimental study, it was found that based on the raw signal root mean square (RMS) levels, the AE technique was more sensitive for fault detection purposes. However, in their experiments, the AE sensors were attached directly on the faulty gear inside the gearbox, which is infeasible for most real applications. Baydar and Ball [21] used the smoothed pseudo-Wigner-Ville distribution to compare the results from acoustic signals and vibration signals. They simulated three types of progressing local faults: broken tooth, gear crack, and localized wear. Their results suggested that acoustic signals are more effective for the early detection of faults and may provide a powerful tool to indicate the various types of progressing faults in gearboxes. However, the acoustic signal presented in their paper was collected by a microphone, which was not exactly acoustic emission. Acoustic emission signal is the elastic stress wave generated inside a solid material, typically metal, due to energy release. Acoustic signal refers to the sound signal which travels in the air and can be collected by a microphone. Acoustic signals are different from acoustic emission signals in that acoustic signals generally lay in the audible range (20 Hz∼20 kHz), while the acoustic emission frequency lies in a higher frequency range (1 kHz∼1 MHz).

Used as a ground reference, reliable AE signals of healthy cases have been acquired by many researchers as an important pre-requisite for the success of AE-based fault detection. In a very recent study on wind turbine condition-based monitoring, a design of a new continuous condition monitoring system with automated warnings based on a combination of vibration and AE analysis was reported in [22]. The authors of the study tried to determine a ground reference for the healthy turbine. The vibrational and AE signatures for a healthy wind turbine gearbox and generator were obtained as a function of wind speed and turbine power. They listed a number of limitations in current research of AE on rolling elements diagnostics. First of all, the measurements are mostly performed on laboratory test rigs other than under field service conditions. Second, the signal to noise ratio is low due to the short duration of data collection. Third, classification algorithms such as pattern recognition could possibly cancel the coherent elements of the noise but not the random or quasi-random components. Thus, they proposed that in order to address the above limitations, future AE work using much longer monitoring times and repeated measurement on actual defect rolling elements in service is needed to compensate for the random noise and instrument performance errors. In another AE-based gear diagnosis paper [23], an energy-based condition indicator was introduced for monitoring and diagnosis under any machine operating conditions in spite of speed and load variations. A feature called energy index (EI) was proposed to measure the statistical relative energy levels of segments in a time domain signal over a cycle. The proposed technique was validated by comparison with some of the existing methods using the same AE data for early fault detection. The proposed method was also tested with vibration data. When applied to AE signals, it was able to effectively detect early faults. However, in their research, AE signals were sampled at a high rate of 1 MHz, which hindered them from doing time synchronous averaging due to the large data volume. They used an alternative method of plotting the result of each revolution together to get a visual data graph of the results. Also, their work aimed to evaluate AE and vibration for fault detection purposes other than fault level diagnostics.

In general, AE-based techniques require much higher sampling rates than vibration analysis-based techniques for gearbox fault diagnosis. In a recent paper, Qu *et al*. [24] proposed a new AE-based gearbox fault diagnostic approach. Their proposed approach combines a heterodyne-based frequency reduction technique with time synchronous average (TSA) and spectral kurtosis (SK) to collect AE signals with a sampling rate that is comparable to that of vibration sensors, processes AE sensor signals, and extracts features as condition indictors for gearbox fault detection. They have shown that the proposed AE sensor-based approach gave good gear fault diagnostic results. However, it is questionable whether an AE-based technique would give a better or at least the same performance as the vibration analysis-based techniques using the same sampling rate. To answer the question, this paper presents a comparative study for gearbox tooth damage level diagnosis using AE and vibration measurements with the same sampling rate. Partial tooth cut faults are seeded in a gearbox test rig and experimentally tested.

## 2. Gear Mechanics Background: Backlash, Contact Ratio, and Tooth Cut

Gear conjugating involves several kinds of stress, among which two basic stress are: contact stress and root bending stress [25,26]. Excessive contact stress causes surface pitting/wear, while the latter causes tooth breakage or tooth root cracks. Backlash and contact ratio can be considered as two major factors contributing to excessive contact stress and the gear noise. They determine the smoothness of the gear meshing and therefore cause the vibration and acoustic emission.

Backlash, in the context of gears and gear trains, is the amount of clearance between mated gear teeth. It is the gap that can be seen when the direction of movement is reversed and the slack or lost motion is taken up before the reversal of motion is complete. The presence of backslash has a significant effect on impact dynamics of meshing gear teeth-pair. Backlash is essential for the gear transmission in the sense that too little backlash may result in interference between the teeth while excessive backlash would cause looseness during gear mating. Backlash has been discussed in gear papers [27–30]. Generally, larger backlash would generate higher gear noise, which is the source of AE and vibration signals.

**Figure 1.**
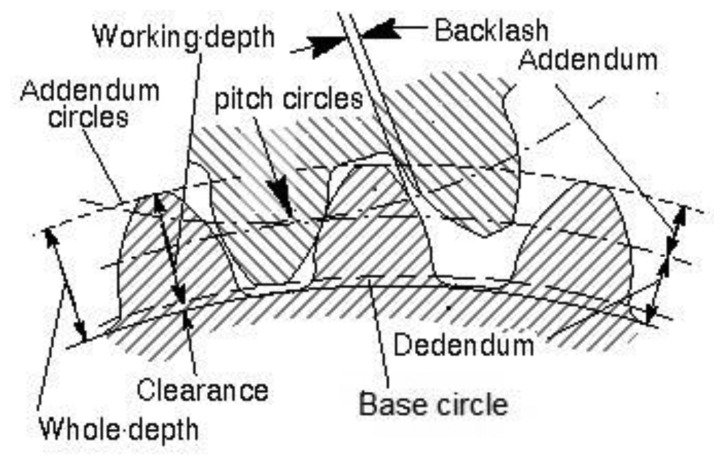
Schematic diagram of two gears meshing.

In order to understand the actual effect of the varying tooth conditions on the gear meshing activity, it is important to take a brief look at the gear profile before and after the tooth cut. The schematic diagram of two gears meshing is shown in Figure 1.

The relationship between gear noise and contact ratio has also been discussed by Tuma [31]. He concluded that high contact ratio of gears is an important factor for gearbox noise reduction. Contact ratio is defined as the number of angular pitches through which a tooth surface rotates from the beginning to the end of contact. In a simple way, it can be defined as a measure of the average number of pairs of teeth in contact during the period in which a tooth comes and goes out of contact with the mating gear. It can be calculated as:

(1) $$\text{Contact}\;\text{ratio}=\;\frac{\sqrt{r_{a1}^{2}-r_{b1}^{2}}+\sqrt{r_{a2}^{2}-r_{b2}^{2}}-\text{Csin}\phi }{P_{c}\text{cos}\phi }$$

where, r_a1_ and r_b1_ are addendum radius (distance from the tops of the teeth of a gear to the gear center) and base radius (distance from the base circle to the gear center) for the pinion gear center, and r_a2_ and r_b2_ are addendum radius and base radius from the pairing gear center, respectively; *C* is the gear axis center distance; *ϕ* is the angel of the pressure line; *P_c_* is the circular pitch of the pinion gear. Circular pitch is length of the arc of the pitch circle between the centers or other corresponding points of adjacent teeth. For more details of the concept and calculation, readers may refer to [32].

From Equation (1), it can be inferred that as the tooth cut gets deeper, the term ${\text{r}}_{\text{a}1}^{2}-{\text{r}}_{\text{b}1}^{2}$ keeps decreasing until it reaches 0 when the tooth cut approaches the base circle. That is, as the gear tooth cut gets deeper, the local contact ratio of the gear gets smaller. As the contact ratio decreases, the amount of meshing looseness increases, which is expected to generate larger gear noise.

In addition, if a tooth was cut deeper than the addendum circle, the tooth would lose the initial contact point during gear meshing and lead to more backlash. For the most common involute gears, the ratio between addendum and dedendum is 1:1.157. Therefore, a 50% depth tooth cut would introduce larger backlash than healthy gear and a 25% tooth cut. A 100% tooth cut would further cause even larger backlash during gear meshing. In short, the deeper the tooth cut, the smaller the contact ratio, and the larger the backlash.

## 3. Gearbox Fault Diagnosis using AE and Vibration Sensors

In this paper, the diagnostic performance of AE sensor- and vibration sensor-based techniques is investigated and compared on a set of seeded gear tooth cut fault test data collected using the same sampling rate. Before the results are presented, both the diagnostic techniques using AE sensors and vibration sensors are explained in this section.

### 3.1. AE Based Gear Fault Diagnosis

#### 3.1.1. The Heterodyne Technique

**Figure 2.**
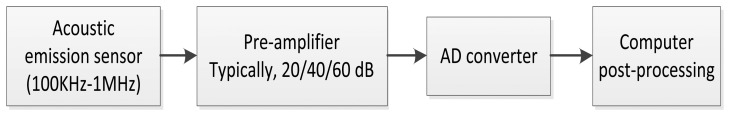
Traditional AE signal acquisition and preprocessing procedure.

To explain the AE-based gear fault diagnostic approach using a comparison, the traditional AE signal processing procedure is first presented in Figure 2.

In a traditional AE signal processing procedure, all of the data is collected and stored on a computer without any signal processing. There are two disadvantages associated with this procedure. First, it increases the data acquisition cost. Second, it relies on the computer to process the resulting large data set. A heterodyne-based frequency reduction technique has been proposed in a previous paper [24]. For the purpose of explanation, the basic principles of heterodyne-based frequency reduction technique are introduced next.

For rotating machinery, a periodic displacement (which may only cause a small acceleration) can be an indication of a fault. The displacement will cause a dislocation associated with the AE signature. The information contained in the AE signature is related to the modulation rate of the signature. This information can be recovered through a demodulation process. The demodulation process is similar to information retrieval in an amplitude/phase modulated radio frequency signal. The carrier signal of a typical AM radio signal is several MHz, while the information modulated onto that signal is an audio signal of a couple of kHz. After demodulating the carrier using an analog signal conditioning circuit, the acquisition system can then be sampled at audio frequency (10 s of kHz). This signal processing can then be performed at lower cost with an analog circuit in comparison with using a high speed analog to digital converter and the associated computation power required to process the large data set as a result of a high sampling rate.

The AE signal demodulator implemented in this paper work similarly to a radio quadrature demodulator: shifting the carrier frequency to baseband, followed by low pass filtering. The technique is called heterodyne. Mathematically, heterodyning is based on the trigonometric identity. For two signals with frequency *f*_1_ and *f*_2_, respectively, it could be written as:

(2) $$\text{sin}(2\pi f_{1}t)\text{sin}(2\pi f_{2}t)=\frac{1}{2}\cos[2\pi (f_{1}-f_{2})t]-\frac{1}{2}\cos[2\pi (f_{1}+f_{2})t]$$

where, *f*_1_ is the carrier frequency, *f*_2_ is the reference input signal frequency of the demodulator.

**Figure 3.**
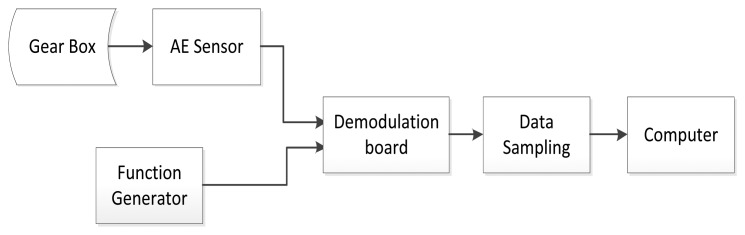
The AE signal acquisition and preprocessing procedure.

It is worth mentioning that the heterodyne technique is aimed at demodulating the amplitude modulated signals other than phase or frequency modulated signals from the raw AE signals. Although frequency modulation and phase modulation could present in the raw AE signals potentially, they are assumed to be trivial and will not be considered. The diagram of the proposed down sampling system using heterodyne is shown in Figure 3.

By adding a demodulation step, the purpose of reducing the signal frequency to tens of kHz could be achieved. This is close to the frequency range of general vibration signals. Any data acquisition board with a low sampling rate could be able to sample the pre-processed AE data.

A key to the success of applying the heterodyne technique to AE signals is to select the right frequency of the reference signal. In this paper, an optimization procedure is developed to search for the optimal frequency of the reference signal using a linear chirp function as the demodulation input. In a linear chirp, the instantaneous frequency *f*(*t*) varies linearly with time. A linear chirp function could be described as:

(3) $$f(t)=f_{0}+k\times t$$

where *f*_0_ is the initial frequency, *k* is the chirp rate, *f*(*t*) is the instantaneous frequency at time *t*.

In searching for the optimal reference frequency, normally a frequency range is pre-selected, for example, 50 kHz–1,050 kHz. The chirp function will start with an initial frequency of *f*_0_ and chirp with a constant rate of *k*. Before the presentation of the algorithm, the following terms are defined:

*f*_min_ = lowest reference frequency
*f*_max_ = highest reference frequency
Δ *f* = frequency increment
$n=\frac{f_{\text{max}}-f_{\text{min}}}{\Delta f}$, the total number of frequency segments
*N_i_* = number of digitized data samples in each segment *i*, *i* = *1,* …, *n*
*X*(*j*) = digitized modulated signal of *x*(*t*), where $x(t)=\frac{1}{2}\cos[2\pi (f_{1}-f_{2})t]$ as derived from Equation (2)
f^*^ = the optimal demodulation reference frequency

The optimization process is to search for the best frequency such that the RMS of the demodulated signal is maximized. It is defined by the following algorithm.



**Algorithm**: Optimal AE reference frequency searching procedure
Step 1. Set the initial frequency *f*_0_ = *f*_min_Step 2. For *i* = 1 to *n*
$$RMS_{i}=\sqrt{\sum_{j=1}^{N_{i}}\frac{X{\left(j\right)}^{2}}{N_{i}}}$$ End For
$i^{*}=\underset{1<i<n}{\text{arg}\;\text{max}}\;RMS_{i}$Step 3. Compute optimal reference frequency of demodulation as: f^*^ = *f*_0_ + i^*^ × Δ*f*


#### 3.1.2. Time Synchronous Averaging

TSA has been widely used in processing the vibration signals for rotating machine fault diagnosis [33–36]. The idea of TSA is to use the ensemble average of a raw signal over a certain number of revolutions in order to enhance signals of interest with less noise from other sources. For a signal function *x*(*t*), being digitized at time intervals *nT* will result in samples *x*(*nT*), where *T* is the sampling interval. Denoting the averaged period by *mT* and the total number of averaged periods by *N*, TSA is given as [36]:

(4) $$y(nT)=\frac{1}{N}\sum_{r=0}^{N-1}x(nT-\text{rmT})$$

More details about TSA could be found in [33].

The successful application of TSA in vibration signal analysis provides opportunities for processing AE signals. Basically, two types of TSA algorithms are available in the literature, *i.e.*, TSA with tachometer, and tachometer-less TSA. In comparison with TSA with tachometer, tachometer-less TSA needs to estimate the angular information from the vibration data. For slow speed variation cases, time domain features like gear meshing information could be used. However, tachometer-less TSA will introduce more phase reference errors and thus have less accuracy than TSA with tachometer. For a complete discussion of order tracking with or without tachometer, please refer to [37]. In this paper, TSA with tachometer is used.

Despite of the popular applications of TSA to vibration signal analysis, applications of TSA to AE signal processing for gear fault diagnosis have not been reported in the literature. The complicated feature and large data volume of AE signals make it unrealistic to execute TSA algorithms directly on AE data. TSA enables the direct comparison of the vibration/acoustic signals produced by each tooth on the same gear over one revolution. TSA for gear diagnosis generally computes the vibration/acoustic signals of a single shaft revolution. After TSA is calculated, basically all kind of fault detection condition indicators can be evaluated on the TSA signal.

#### 3.1.3. AE Condition Indicators

There are many condition indicators available in literature. Most of the condition indicators deal with the data distribution, such as peakiness, amplitude level, deviation from the mean and so on. A brief introduction of the condition indicators applied to AE signals is given next.

RMS: The root mean square for a discretized signal is defined as:

(5) $$x_{\text{rms}}=\sqrt{\frac{1}{N}\sum_{i=1}^{N}\left({x_{i}}^{2}\right)}$$

where, *x_rms_* is the root mean square value of dataset *x*, *x_i_* is the *i*th element of *x*, *N* is the length of dataset *x*.

P2P: Peak to peak value of a dataset *x* is defined as:

(6) $$P2P=\frac{\text{Max}(x)-\text{Min}(x)}{2}$$

where, *Max* (*x*) is the maximum value of *x*, *Min* (*x*) is the minimum value of *x*.

Kurtosis: kurtosis describes how peaky or how smooth of the amplitude of dataset *x* is. If a signal contains sharp peaks with high values generated by a fault in the gearbox, it is expected that its distribution function will be sharper. Thus, the kurtosis of the fault signal should be higher than that of the healthy signal. The function of kurtosis is given as:

7 $$\text{Kurt}=\frac{N\sum_{i=1}^{N}{\left(x_{i}-\bar{x}\right)}^{4}}{{\left[\sum_{i=1}^{N}{\left(x_{i}-\bar{x}\right)}^{2}\right]}^{2}}$$

where *Kurt* is the kurtosis of dataset *x*, *x_i_* is the *i*th element of *x*, *N* is the length of dataset *x,x̄* is the mean value of dataset *x*. It is worth mentioning that for any normal distribution, the kurtosis value is 3. This could be easily verified by the moment generating function.

In addition to the condition indicators computed directly using the AE TSA signals, RMS of the residual signal can also be computed. A residual signal is generally defined as a synchronous averaged signal with the shaft, gear mesh, and their associated harmonic frequencies removed.

### 3.2. Vibration Based Gear Fault Diagnosis

For vibration signals, a similar process flow is applied except for the heterodyne technique which is not needed for vibration signals. After TSA, different condition indicators are computed for vibration signals. Basically, the vibration signal frequency is more closely related to gearbox rotational frequency and mechanical interaction. In addition to RMS and P2P, two physical meshing behavior related condition indicators: FM0 and SLF are introduced next.

FM0: FM0 is the zero-order figure of merit. It is a global indicator that reacts to changes in the whole frequency range of the average and identifies major abnormal behaviors with regard to meshing pattern. FM0 is defined as the ratio of peak to peak amplitude (PPA) of the TSA signal to the sum of amplitudes of gear mesh frequency and its harmonics. An increase in peak to peak level is generally observed in case of major tooth faults such as tooth breakage without significant change in the mesh frequency, which will result in increase of FM0 value [38]. FM0 will increase if a periodic signal contains a local increase in amplitude. Mathematically, it is expressed as follows:

(8) $$FM0=\frac{P2P_{\text{TSA}}}{\sum_{i=1}^{n}A(f_{i})}$$

where, FM0 is the zero-order figure of merit; *P2P_TSA_* is the peak to peak value of the vibration TSA in the time domain; *A*(*f_i_*) is the amplitude of the *i*th harmonic of the gear meshing frequency.

SLF: SLF stands for the sideband level factor. It is the sum of the first order sideband amplitudes of the fundamental gear meshing frequency normalized by the RMS of the synchronous time average [39]. SLF is a good indicator of single tooth damage or gear shaft damage. The formula for SLF is given by:

(9) $$\text{SLF}=\frac{R_{I,-1}(x)+R_{I,+1}(x)}{\text{RMS}(x)}$$

where *x* is the vibration signal TSA, *R_I_*_,−1_(*x*) is the amplitude of the first order left-hand side sideband, *R_I_*_,+1_(*x*) is the amplitude of first order of right-hand side sideband. *RMS*(*x*) is the RMS of *x*.

## 4. Experimental Setup

**Figure 4.**
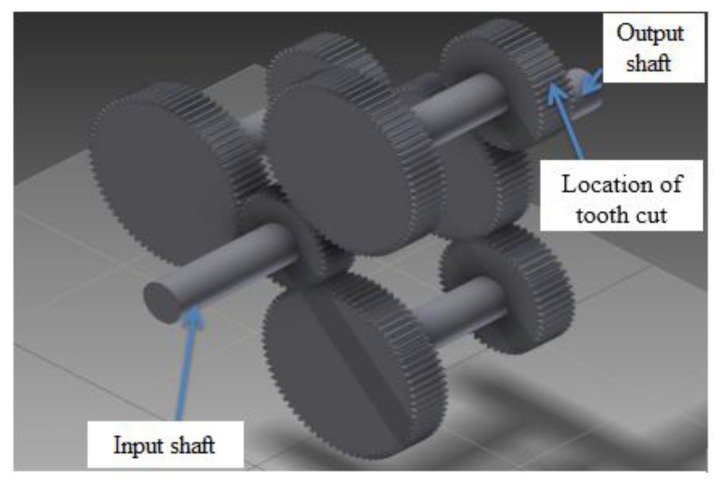
The structure of the notational split torque gearbox.

**Figure 5.**
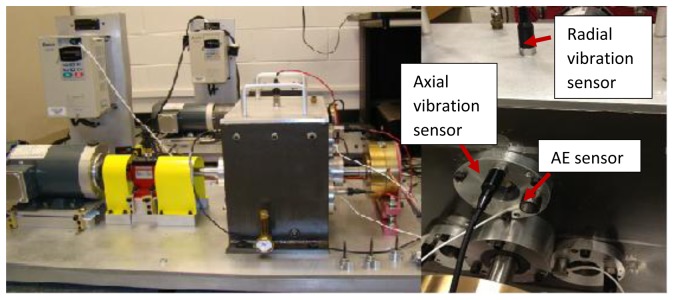
The notational split torque gearbox and sensor locations.

In order to compare the gearbox fault diagnostic performance of the AE and vibration sensors, tests with gear tooth cut seeded faults were conducted on a notational two stage split torque gearbox (STG) in a laboratory. In a STG, there are several identical intermediate gear pairs which could split the torque evenly. Also, the intermediate gear pair could offer a larger transmission ratio. Both the input side and output side of the STG use a parallel shaft layout. All of the gears inside are spur gears. On the input side, the input driving gear is a 40 teeth gear which drives three input driven gear with 72 teeth each. On the output side, three output driving gears with 48 teeth drive a 64 teeth gear. A 3 HP three phase induction AC motor with a maximum speed of 3,600 rpm is used to drive the notational gearbox. To accommodate for shaft misalignment and reduce the vibration transmission, a disc type coupling is utilized to transmit the torque from the motor to the driving shaft. A magnetic loading system is controlled by a power supply and the load can be adjusted by changing the output current of the power supply. Figure 4 shows the structure of the notational split torque gearbox. The test rig and sensor locations are shown in Figure 5.

As a speed reduction gearbox, the input side and the output side have a 2.4 times speed reduction ratio. Based in the input speed tested in the experiments, the corresponding output shaft speed and intermediate shaft (faulty gear shaft) speed is provided in Table 1.

**Figure 6.**
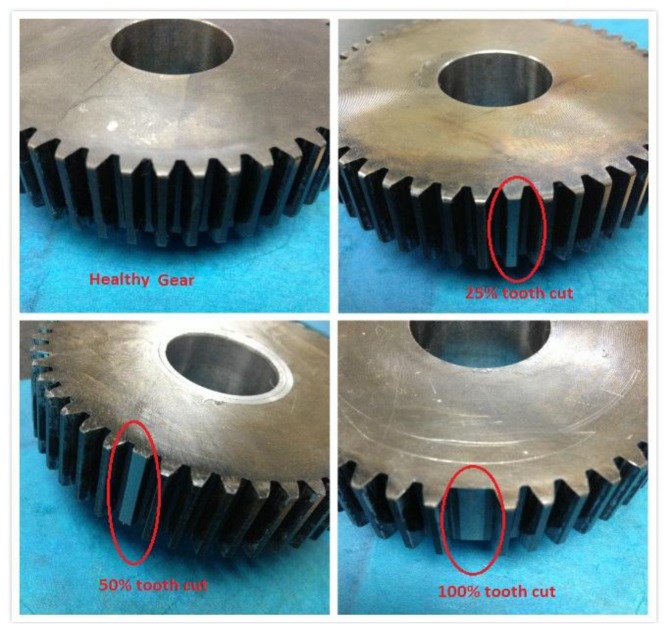
Seeded tooth cut faults.

For the faulty gearbox, one of the intermediate gears with 48 teeth on the output side was damaged by artificially cutting a tooth by a certain percentages of the tooth depth. As shown in Figure 6, 25%, 50%, and 100% tooth cuts were created, respectively. The fault created here is relatively large, but the tooth cut did not cause any severe failure to the gearbox because of the split torque feature. The slack or loss of motion due to the tooth cut or tooth missing on one of the driving pinion gears can be compensated by the other two driving pinion gears.

One AE sensor and two accelerometers were mounted on the gearbox. The AE sensor was attached to the gear housing using adhesives as shown in Figure 5. One accelerometer was mounted on the gearbox housing in the axial direction and the other one was mounted on top of gearbox housing in the radial direction (see Figure 5). The signals from all of the three sensors were collected simultaneously during the test runs. In addition, tachometer signals were collected along with vibration and AE signals.

For AE data acquisition, a true differential wideband sensor with high sensitivity and bandwidth was used. It has a good frequency response over the range of 100–900 kHz. Differential sensors offer a lower noise output from a pre-amplifier. The accelerometers used for vibration data collection were the industrial ICP accelerometer model No. IMI 608-A11. The frequency response of the accelerometers is from 0.5 Hz–10 kHz. The heterodyne process was accomplished by a hardware demodulation. A demodulation board (Analog Devices-AD8339) and a sampling device (NI-DAQ 6211) were used. The demodulation board performed the multiplication of sensor signals and reference signals. The demodulation board is an analog device and much more affordable than a high sampling rate data acquisition board. It takes two inputs, one from the AE sensor, and the other from function generator as a reference signal. The basic principle of AD8339 could be explained by Gilbert cell mixers. In electronics, the Gilbert cell is commonly used as an analog multiplier and frequency mixer. The output current of this circuit is an accurate multiplication of the base currents of the both inputs. According to Equation (4), it could convert the signals to baseband and twice the carrier frequency. The frequency of reference signal was obtained as 400 kHz by the optimization algorithm described in Section 3.1. In searching for the optimized reference frequency, a chirp function with a range of 50 kHz–1,050 kHz was selected to cover the whole sensor response range. The chirp function started with an initial frequency of 50 kHz and chirp up at a rate of 139.89 kHz/s. The output of the demodulation board goes to the sampling board and the high frequency component is filtered out. NI-DAQ 6211 is a low speed data acquisition device with a sample rate up to 250 kS/s.

For signal acquisition, Labview signal express software was used. During the experiments, continuous AE signals were collected. The data sampling rate was set to 100 kHz for both vibration and AE signals in order to make a fair comparison. Zero loading condition was applied throughout the test. The gearbox was run with six different input shaft speeds starting from 10 Hz and increased in 10 Hz increments to 60 Hz. For each speed, five data sets were collected. In order to get a good TSA result, signals were recorded over approximately 200 revolutions.

## 5. Results and Discussions

In this section, the diagnostic results of the gear seeded cut fault tests using both AE and vibration sensors are provided and discussed.

### 5.1. Results of AE Signal Analysis

After heterodyning, TSA was performed on the signals first to get the TSA signals using the tachometer signal as the phase reference. Then the AE signal condition indicators were calculated on the TSA signals. Three condition indicators as introduced in Section 3.1.3, were computed using the AE TSA signals: RMS, P2P and kurtosis. In addition, RMS values of the residual signals were also computed for comparison.

**Figure 7.**
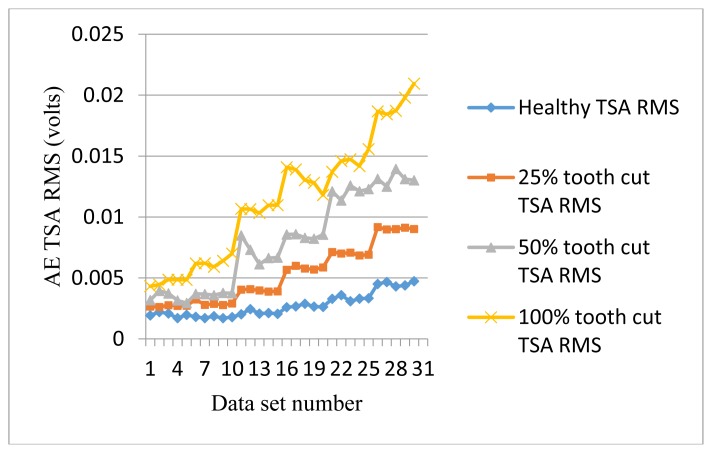
RMS of AE TSA signals.

Figure 7 shows the RMS plots of AE TSA signals. The data set numbers were arranged from 10 Hz–60 Hz, five data sets for each speed. Other plots in the following context are arranged in the same manner. It can be seen from Figure 6 that the RMS AE TSA signals provided a good trend for the energy level with the increase of speed. For different levels of the tooth cut, it offers clear separation. This result shows that AE signals are very sensitive to the gear meshing impact due to both speed and level of severity of tooth cut fault.

**Figure 8.**
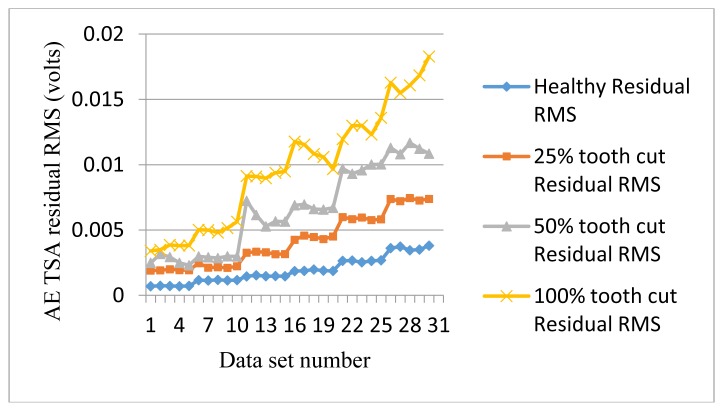
RMS of AE residual signals.

Figure 8 shows the RMS plots of AE residual signals. Residual RMS provides similar separation as the AE TSA RMS but reduces the degree of fluctuation. It also increases the fault detectability between healthy and 25% tooth cut fault since the separation between the two is bigger. As residual signals normally contain the fault features except gear meshing and harmonics, it could be more effective than the TSA signal itself.

**Figure 9.**
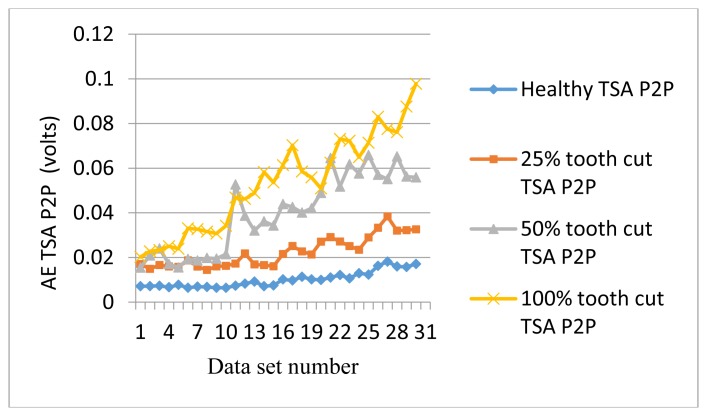
P2P of AE TSA signals.

**Figure 10.**
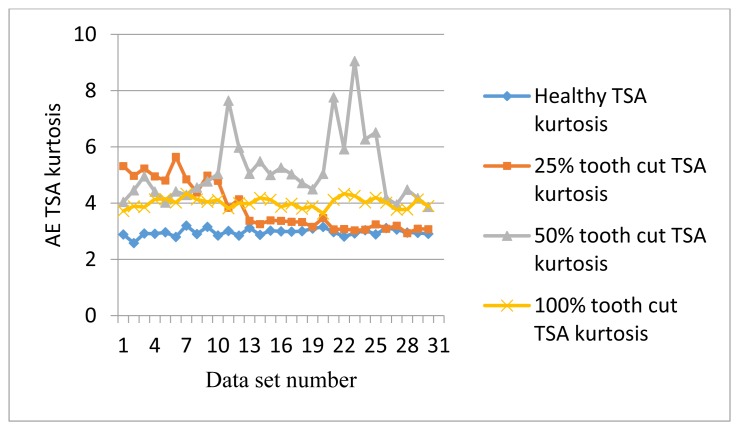
Kurtosis of AE TSA signals.

Figure 9 shows the P2P plots of the AE TSA signals. As can be seen from Figure 9, P2P generally follows the trends with the increase of the speed but contains some more fluctuation compared with RMS. Figure 10 shows the kurtosis plots of AE TSA signals. Although kurtosis is not able to distinguish fault levels, it acts as a good condition indicator for fault detection. For any Gaussian distribution, the value of kurtosis is calculated as 3. As can be seen from Figure 10, all of the healthy signal kurtosis values are close to 3. On the other hand, the faulty signal kurtosis values are mostly above 3. Since kurtosis is not affected by the speed, it is useful for making fault detection decisions.

### 5.2. Results of Vibration Signal Analysis

**Figure 11.**
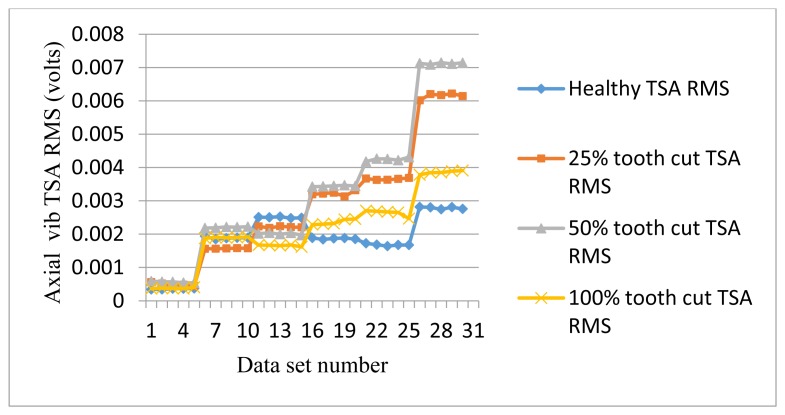
RMS of axial vibration TSA signals.

**Figure 12.**
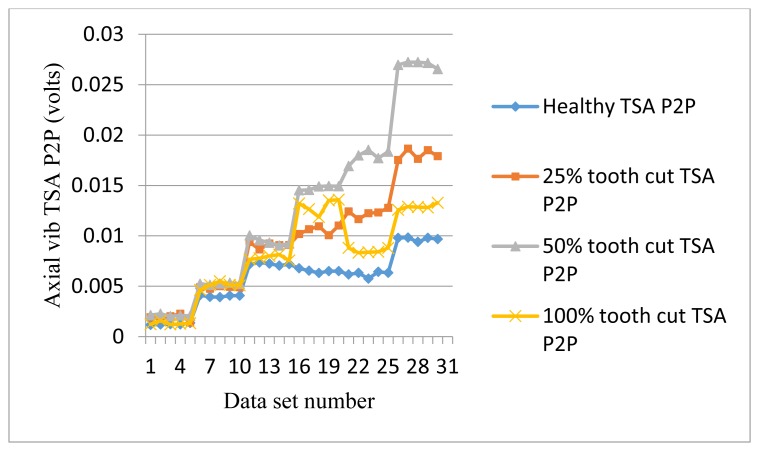
P2P of the axial vibration TSA signals.

**Figure 13.**
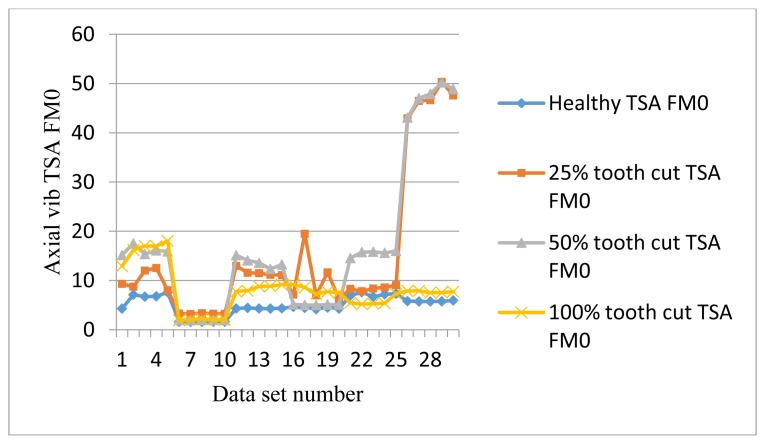
FM0 of the axial vibration TSA signals.

**Figure 14.**
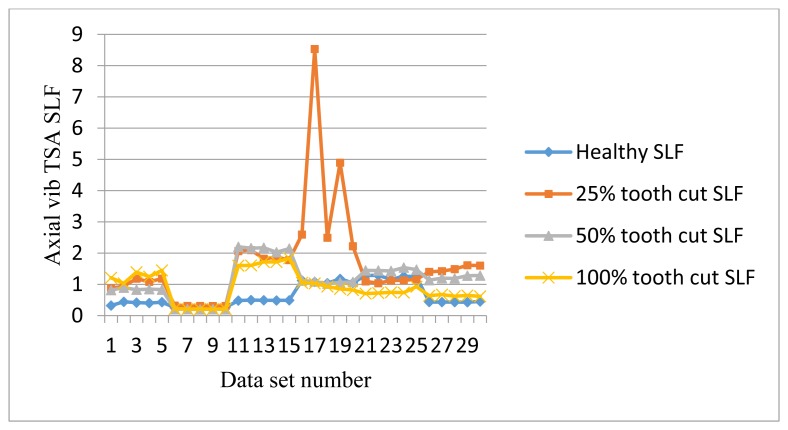
SLF of axial vibration TSA signals.

**Figure 15.**
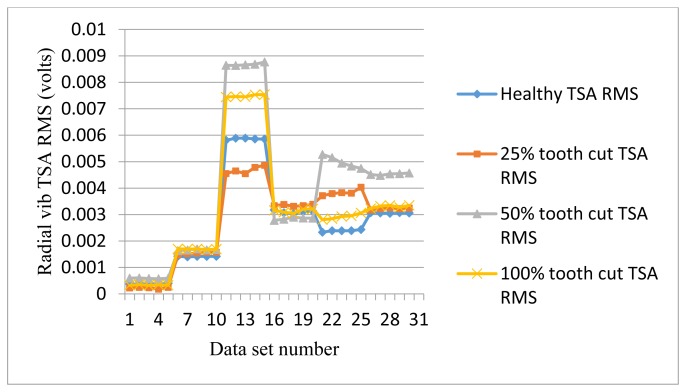
RMS of radial vibration TSA signals.

**Figure 16.**
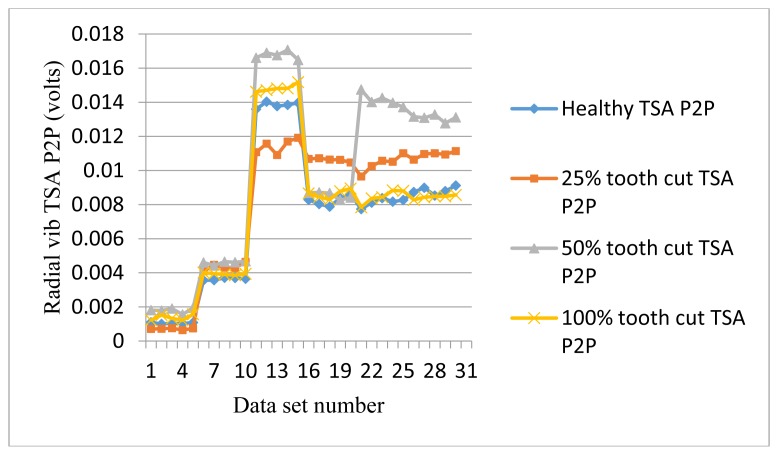
P2P of radial vibration TSA signals.

The frequency response range of vibration signals is much lower than that of AE signals. Therefore a vibration signal has the advantage of representing the mechanical behaviors more closely, but it also has the disadvantage of being easily effected by mechanical resonance. Like AE signal processing, TSA was performed on raw vibration signals first, then the condition indicators were computed. A total of 4 condition indicators were computed for vibration signals: RMS, P2P, FM0, and SLF. During the experiments, both axial and radial direction vibration signals were collected and analyzed. Figures 11, 12, 13 and 14 show the results from the axial direction vibration sensor. Figures 15, 16, 16 and 18 give the results from the radial vibration sensor.

Figure 11 shows the RMS plots of the vibration TSA signals. Note that the sensors used in the experiments have a sensitivity of 100 mV/g. Based on this rate, the voltage unit on the vertical axis could be converted to *g* units by multiplying by a factor of 10. It can be seen that in the low speed range below 30 Hz, the vibration TSA RMS does not give any indication of the fault. In the high speed range above 30 Hz, vibration TSA RMS with tooth faults increases significantly and provides good indication for fault detection. However, vibration TSA RMS is not sensitive to the level of tooth cut as the vibration TSA RMS for 100% cut is lower than that of 50% and 25% tooth cut.

Figure 12 gives the P2P plots of vibration TSA signals. P2P values of the faulty signals are mostly higher than the healthy counterpart, except at 10 Hz input speed. Like RMS, P2P shows potential capability for fault detection, but not for fault level diagnostics.

Figure 13 shows the FM0 plots of the axial vibration signals. FM0 could detect the anomalies in most of the cases. However, it has a lot of fluctuation at different speeds. Again it is not effective for damage level separation.

Figure 14 shows SLF plots of the axial sensors. It can be seen that at 10 Hz, 30 Hz, and 60 Hz, the healthy SLF is lower than all faulty ones. But for the other speeds, some of the faulty signal SLFs are lower than healthy one. This result shows that SLF of axial vibration is not effective for fault detection.

Figure 15 shows the RMS plots of the radial vibration TSA signals. It can be seen from Figure 15 that the radial vibration signals are seriously affected by the mechanical resonance, especially at 30 Hz. Basically, the RMS of radial vibration signals does not give a good indication for gear tooth cut faults and the level of the cut.

**Figure 17.**
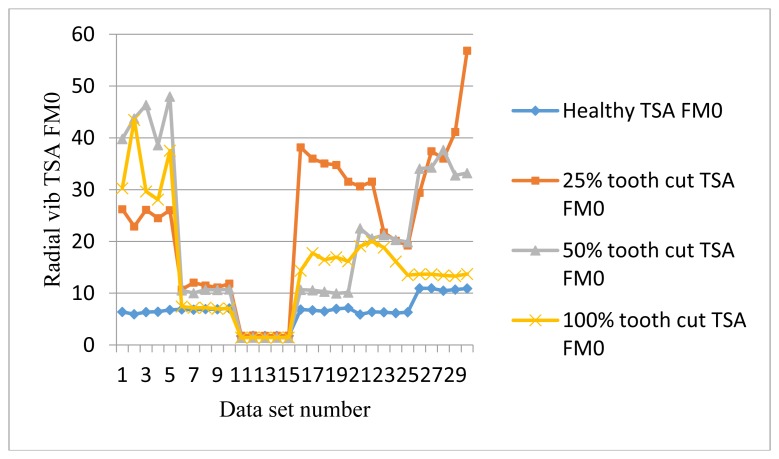
FM0 of radial vibration TSA signals.

Similarly, the P2P of the radial vibration signals does not give a good indication either, as shown in Figure 16. Figure 17 shows FM0 plots of the radial vibration sensors. It can be seen that except at 20 Hz and 30 Hz, the FM0 of the faulty signals are higher than the healthy ones. At 30 Hz, FM0 for all cases dropped to a very low level and overlapped each other. At the same time, RMS and P2P from Figures 15 and 16 show that the peaks and energy levels for all cases increased significantly. It is likely to be caused by mechanical resonance. Other than that, the healthy FM0 is relatively stable with a bound of approximately 10, while the faulty signals can go as high as 40, which makes FM0 a good condition indicator for tooth cut fault detection.

**Figure 18.**
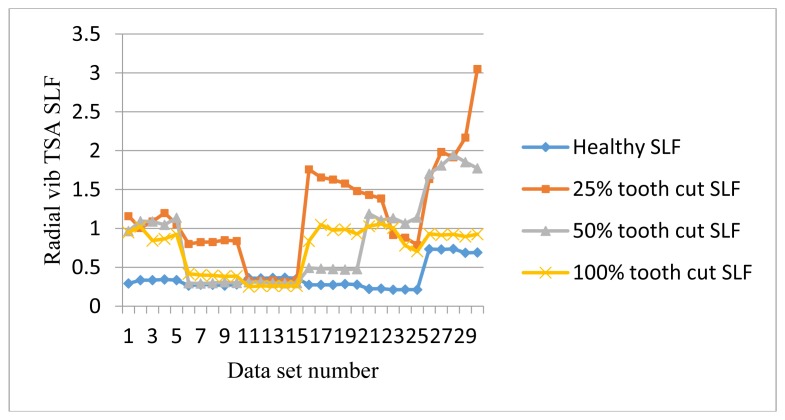
SLF of radial vibration TSA signals.

Figure 18 shows SLF plots of radial vibration sensors. SLF works like FM0. It could clearly separate the healthy signals from the faulty ones under the low speed of 10 Hz as well as the high speed of 30 Hz and above, but it fails to distinguish the faults at 20 Hz and 30 Hz.

In summary, it can be seen that for axial vibration sensor mounted on the bearing housing, the RMS and P2P show good fault detection potential. FM0 and SLF of the axial sensor work in most cases but are not stable. On the other hand, for the radial sensor mounted on the top of the gearbox housing, RMS and P2P fail to work, while FM0 and SLF work for fault detection purposes. Compared with AE results, none of the vibration condition indicators could detect the tooth cut level. The vibration signals are highly affected by background noise or mechanical resonance, making their performance unstable. AE RMS and P2P show a roughly linear relationship with shaft speed. They could clearly indicate the tooth cut levels for diagnostics. Also, kurtosis of AE signals offers another effective index for fault detection. It should be emphasized here that given the frequency range of 0.5–10 kHz of the vibration sensors used in the experiment, the vibration signals collected at a sampling rate of 100 kHz were still considered as oversampled.

It is also necessary to point out that vibration signals offer better frequency domain resolution. Since both FM0 and SLF are calculated based on gear meshing frequency and considered as frequency domain condition indicators, FM0 and SLF computed using vibration signals give better performance than other time domain condition indicators such as RMS and P2P. However, FM0 and SLF computed using AE signals were not as good as RMS and P2P computed using AE signals. Therefore, they are not included in the AE results.

As explained in Section 2, the tooth cut fault is the direct cause of larger backlash and reduction in contact ratio. Both the large backlash and low contact ratio introduce more looseness during gear meshing and therefore cause higher impact and gear noise. From this perspective, it can be inferred that AE sensors are much more sensitive to impact energy. Vibration measured by accelerometer is the acceleration signal, which is less sensitive to direct impact energy.

In analyzing the vibration data, in an attempt to remove noises from the vibration signals before the TSA signals were computed, different types of filters were tested. Among them, the best two filters were selected. The first filter was a low pass filter with a cut-off frequency of 10 kHz since the vibration sensor response range is 0.5 Hz–10 kHz. The second filter was a band pass filter with a band width of 1 kHz–10 kHz which would filter out most of the low frequency mechanical background noise as well as high frequency noise. Both filters were designed using a zero-phase shifting filter which would not affect the accuracy of TSA. However, the results with the filters were not significantly different from those obtained without filtering.

It should be emphasized that the results presented in this paper were obtained in a laboratory test rig. It is expected that the level of environmental noise in an actual application could be higher than in a laboratory. The effect is that AE signals have a lot of noises. However, the proposed methodology validated in a laboratory environment should work as well in an actual application. First, AE sensors are known to be less sensitive to background noise and mechanical resonance. Second, the TSA method is a well-developed noise reduction technique. It could significantly increase the signal to noise ratio for the AE signals through synchronization, especially for gear structures.

## 6. Conclusions

Previous research has showed that AE sensor-based approaches using a sampling rate that is comparable to that of vibration analysis gave good gear fault diagnostic results. However, it is questionable whether an AE-based technique would give a better or at least the same performance as the vibration analysis-based techniques using the same sampling rate. To answer the question, this paper presented a comparative study for gearbox tooth damage level diagnostics using AE and vibration measurements. Three different levels of tooth cut faults were artificially created and tested on a notational split torque gearbox in a laboratory. For the AE-based gear fault diagnostic approach, a hardware frequency convertor based on heterodyne technique was used for AE data collection. Both the AE signals and vibration signals were collected with the same sampling rate of 100 kHz. Time synchronous averaging was applied to both types of signals. Condition indicators were then calculated respectively for AE and vibration signals. Experimental results were provided and explained. Based on the experimental results, the following conclusions can be drawn:

1. AE signals could be sampled at 100 kHz while maintaining the capability of distinguishing tooth damage levels using TSA RMS and P2P.
2. AE signals are insensitive to mechanical background noise and mechanical resonance. Therefore, AE signals have the potential to provide better condition indicators for gear fault diagnosis.
3. Vibration signal condition indicators are not consistent with gear tooth damage level. Vibration is less sensitive than AE to small tooth damage in the low speed range.

## Figures and Tables

**Table 1.** Output shaft speed corresponding to input shaft speed.

**Input shaft speed (Hz)**	10	20	30	40	50	60
**Faulty gear shaft frequency (Hz)**	5.56	11.1	16.7	22.2	27.8	33.3
**Output shaft speed (Hz)**	4.17	8.33	12.5	16.7	20.8	25
**Output side gear meshing frequency (Hz)**	266.7	533.3	800	1,066.7	1333.3	1,600
